# The contribution of travel-related urban zones, cycling and pedestrian networks and green space to commuting physical activity among adults – a cross-sectional population-based study using geographical information systems

**DOI:** 10.1186/s12889-016-3264-x

**Published:** 2016-08-11

**Authors:** Tomi E. Mäki-Opas, Katja Borodulin, Heli Valkeinen, Sari Stenholm, Anton E. Kunst, Thomas Abel, Tommi Härkänen, Leena Kopperoinen, Pekka Itkonen, Ritva Prättälä, Sakari Karvonen, Seppo Koskinen

**Affiliations:** 1 National Institute for Health and Welfare (THL), Helsinki, Finland; 2 Department of Public Health, University of Turku, Turku, Finland; 3 University of Amsterdam, Academic Medical Centre (AMC), Amsterdam, The Netherlands; 4 University of Bern, Institute for Social and Preventive Medicine, Bern, Switzerland; 5 Finnish Environment Institute (SYKE), Helsinki, Finland; 6 City of Helsinki, Helsinki City Rescue Department, Helsinki, Finland

**Keywords:** Transport-related physical activity, Green space, Built environment, Socioecological model, Population study, GIS, Finland

## Abstract

**Background:**

The current political agenda aims to promote active environments and physical activity while commuting to work, but research on it has provided mixed results. This study examines whether the proximity of green space and people’s residence in different travel-related urban zones contributes to commuting physical activity.

**Methods:**

Population-based cross-sectional health examination survey, Health 2011 study, and geographical information system (GIS) data were utilized. The GIS data on green space and travel-related urban zones were linked to the individuals of the Health 2011 study, based on their home geocoordinates. Commuting physical activity was self-reported. Logistic regression models were applied, and age, gender, education, leisure-time and occupational physical activity were adjusted. Analyses were limited to those of working age, living in the core-urban areas of Finland and having completed information on commuting physical activity (*n* = 2 098).

**Results:**

Home location in a pedestrian zone of a main centre (odds ratio = 1.63; 95 % confidence interval = 1.06–2.51) or a pedestrian zone of a sub-centre (2.03; 1.09–3.80) and higher proportion of cycling and pedestrian networks (3.28; 1.71–6.31) contributed to higher levels of commuting physical activity. The contribution remained after adjusting for all the environmental attributes and individuals. Based on interaction analyses, women living in a public transport zone were almost two times more likely to be physically active while commuting compared to men. A high proportion of recreational green space contributed negatively to the levels of commuting physical activity (0.73; 0.57–0.94) after adjusting for several background factors. Based on interaction analyses, individuals aged from 44 to 54 years and living in sub-centres, men living in pedestrian zones of sub-centres, and those individuals who are physically inactive during leisure-time were less likely to be physically active while commuting.

**Conclusions:**

Good pedestrian and cycling infrastructure may play an important role in promoting commuting physical activity among the employed population, regardless of educational background, leisure-time and occupational physical activity. Close proximity to green space and a high proportion of green space near the home may not be sufficient to initiate commuting physical activity in Finland, where homes surrounded by green areas are often situated in car-oriented zones far from work places.

## Background

Across Europe, there is currently a political agenda to promote active environments and physical activity at population level [1, 2]. Commuting physical activity, such as walking or cycling to and from work [3, 4], is an environmentally friendly and easily accessible way for most individuals to increase their daily physical activity. However, mixed results or no association have been demonstrated between environmental factors such as green space or pedestrian zones and commuting physical activity [5, 6]. Moreover, scientific information is needed on whether commuting physical activity at population level could be promoted by improving environmental factors.

Cycling and walking are among the most common ways for adult Finns to travel from home to work: 28 % of men and 42 % of women are physically active while commuting (e.g. walk or cycle 15 min or more per day) [7, 8]. According to the latest National Travel Survey, Finns cycle and walk more often in urban than in non-urban settings [9]. In 2010, 10–31 % of Finns lived in a pedestrian zone with sidewalks and pedestrian and cycle lanes, and 26 % lived in a public transport zone [10]. Among employed adult Finns, the average distance from home to work was 14 km in 2010 [11]

Urban areas in the largest Finnish cities consist of 31 to 48 % of green space [12]. The biggest entities of the green space are located on the fringes of cities and include natural forests, grasslands, moors, heathlands, inland marshes and peat bogs, which are important places for outdoor recreation. However, there are urban forests, parks and other green space also inside the densely built areas and, with built walking paths and cycle paths, these can offer a pleasant commuting environment.

The association between the proximity of green space and commuting physical activity has seldom been examined at population level [1]. A high density of green space may not be associated with total physical activity [13], but might be associated with transport-related walking [14] and with recreational walking [15, 16]. The density of green space in a neighbourhood is not necessarily associated with an individual’s actual use of green spaces for recreational walking [17]. On the other hand, a good access to green space can be associated with recreational cycling [18]. A qualitative study reported that some individuals saw green space as suitable for transport-related cycling, while others found it undesirable, unsafe or “not for us” [19]. It should, however, be noted that urban and rural green space in the USA, Canada and Australia, where most of the previous studies have been conducted, is quite different compared to Europe [6, 20]. Many cities in the USA are mainly car-oriented, and the green space coverage is low [21]. These findings cannot, therefore, be simply transposed to the European context when planning for green environments to promote commuting physical activity. Compared to many European countries, Finland has fewer big cities, yet a larger surface area.

The scientific evidence on whether different types of urban areas might be important for commuting physical activity is lacking [1]. Previous studies suggest that street connectivity, pedestrian lanes, and a high density of cycle paths might be positively associated with transport-related walking and objectively measured physical activity [20, 22, 23] as well as with recreational walking and cycling [18, 24]. However, walking and cycling routes might be efficient in promoting transport-related physical activity only among those with no car or inconvenient public transport [25, 26]. More scientific research is, therefore, needed to better understand the role of the different types of urban areas on commuting physical activity in the European context [6, 27].

One should also note that individual factors such as age, gender, education and social support as well as other physical activities during leisure time or at work might influence participation in commuting physical activity [7, 28–30]. Such individual factors, might also influence in which environment one is physically active [5, 6, 23, 31].

To address the gap in scientific knowledge, the aim of this study was to examine how the proximity of green space and living in different travel-related urban zones contribute to commuting physical activity among employed Finns. Individual-level factors, namely age, gender, education, leisure-time and occupational physical activity as well as health and social support, were also taken into consideration as potential background and confounding factors.

## Methods

**Fig. 1:**
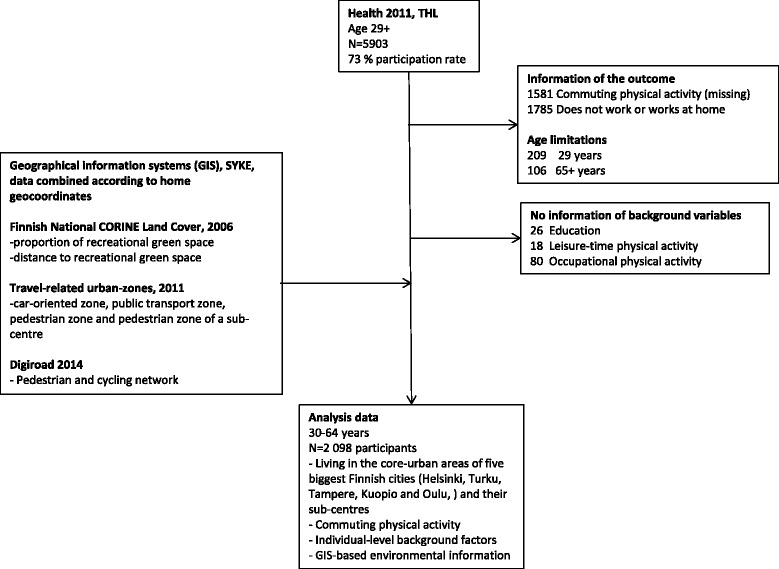
Formation of the study data from Health 2011 data, THL, and Geographical Information system (GIS) datasets

Health 2011 was a health examination survey in Finland that was conducted between August 2011 and June 2012 by the National Institute for Health and Welfare (THL). The cross-sectional study-design involved a representative sample of 8,135 women and men aged 30 years or more, drawn from the national population registers [32]. All participants (*n* = 5,903, participation rate 72.5 %) underwent the following survey protocol: they were clinically examined (including measurements such as anthropometrics, blood pressure, and blood sample as well as functioning test) and interviewed using structured interview (including such as health, illnesses, services, oral health, living habits, functional capacity and working capacity). Participants were also asked to fill out several self-administered questionnaires on health behaviours, health and quality of life. In this study, analyses were limited to those of working age, living in the core-urban areas (in the five largest cities Helsinki, Turku, Tampere, Kuopio, Oulu) and their sub-centres and having completed the information of commuting physical activity and the other examined background variables. The composition of the data for the final analyses, consisting of total *n* = 2098 participants, is described in more detail in Fig. 1.

### Commuting physical activity

Commuting physical activity was measured as the daily amount of walking or cycling to and from work, using the question: “How many minutes do you walk or cycle to and from work daily?” The response alternatives were: 1) “I am not working or I work at home”, 2) “I use public transport or a car for commuting”, 3) “less than 15 min per day”, 4) “15–30 min per day”, 5) “30–60 min per day”, and 6) “over an hour per day”. Participants were instructed to answer questions on physical activity according to following guideline: “If your physical activity changes a lot between the seasons, please choose the alternative that describes best your average situation”. For analyses in this study, commuting physical activity was dichotomised: “active” (responses = 3–6) and “inactive” (=2). Those who had responded “I am not working or I work at home” were excluded from the analyses. Regarding the physical activity measurement reliability, a similar physical activity question was used in the Health 2000 study before [33], and has been found to be a strong predictor of morbidity and mortality [34, 35].

### Distance to and proportion of recreational green space

GIS data was derived from several sources (Table 1). The environmental data was linked to the individuals, based on their home geocoordinates. Green space was extracted from the Finnish national CORINE Land Cover 2006 raster at a 25-metre pixel size. For the analyses, all land cover classes presumably suitable for recreation were extracted from CORINE data [36, 37]: sport and leisure facilities, broad-leaved forests, coniferous forests, mixed forests, natural grass lands, moors and heathlands, transitional woodlands/shrubs, beaches, dunes, sand plains, bare rocks, inland marshes, peatbogs and salt marshes. Only continuous land areas having a minimum size of 1.5 ha were included in the analyses of areas suitable for recreation (hereinafter green spaces) as recommended by the Ministry of the Environment [38]. Euclidean distance from each respondent’s residence to the nearest green space was calculated using ArcGIS 10.1. software [39]. The Euclidian distance to the closest green space from the place of residence was chosen as a proxy for the availability of green space for recreation. As the Ministry of the Environment [38] has set a goal that every Finn should have a recreational green space within 300 metres from their place of residence, the distance to green space was dichotomised as: 1) “0–199 metres” and 2) “200 metres or more”. Moreover, we examined the proportion (%) of recreational green space within 500 meters from home location classified as: 1) 0 = no green space, 2) 1–24,9 % green space, 3) 25–49.9 % of green space and 4) 50 % or more green space.

**Table 1:** Description of environmental variables, values and data sources of GIS-datasets utilized in this study

Environmental variable	Values	Data source, year
Percentage of green areas suitable for recreation (min 1.5 ha)	0–1	Corine Land Cover, 250mx250m, 2006
Euclidean distance to green areas suitable for recreation (min 1.5 ha)	metres	Corine Land Cover raster 25m, 2006
Travel-related urban zones	pedestrian zone, pedestrian zone of a sub-centre, fringe of a pedestrian zone, intensive public transport zone, public transport zone, weak public transport zone and car-oriented zone	Urban Zones classification, 2010
Proportion of motorways	0–1	Digiroad, 250mx250m, 2014
Proportion of cycling and pedestrian lanes	0–1	Digiroad, 250mx250m, 2014
Proportion of roads suitable for cycling and walking	0–1	Digiroad, 250mx250m, 2014

### Travel related urban zones

**Fig. 2:**
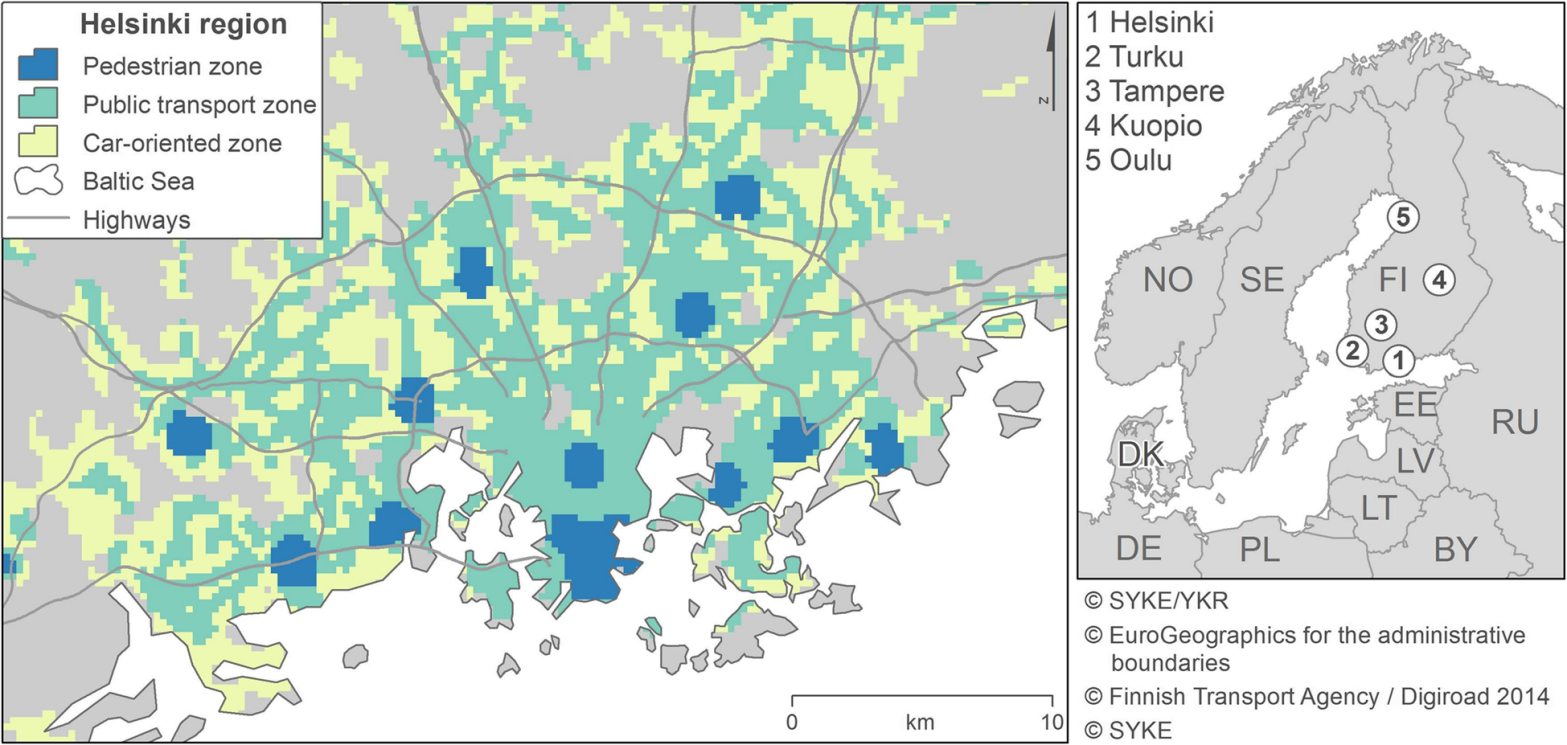
Travel-related urban zones in Helsinki Region (on the left), the five biggest Finnish cities (Helsinki, Turku, Tampere, Kuopio and Oulu) and their surrounding included in the analysis (on the right)ᅟ

Finnish urban regions have been divided into travel-related urban zones based on each spatial unit’s (250 m pixels) location within the urban structure and public transport supply [40]. The types of urban zones area are*: 1) pedestrian zone*, 2) *pedestrian zone of a sub-centre*, 3) *fringe of a pedestrian zone*, 4) *intensive transit zone*, 5) *transit zone*, 6) *weak transit zone* and *7) car-oriented zone*. The approach takes the polycentrism of urban regions into account by also identifying urban sub-centres. The criteria of the travel-related urban zones are based on distance from the central business district (CBD), distance from a public transport stop, headway of public transport and location of sub-centres. Pedestrian zone is limited from 1 to 2 km radius of CBD area, surrounded by a fringe of a pedestrian zone in a radius of 2 to 5 km from the CBD. In large urban areas, the sub-centres have their own independent pedestrian zones. Public transport zone has a criterion of bus traffic having headway from 5 to 15 min at high rush hours in urban areas and in other non-urban areas a headway of 15 to 30 min. Car-oriented zones are, in general, located in the outskirts of urban areas, where the population density is too low to organise frequent public transport. Participants living outside travel-related urban zones and living in rural areas were in this study classified in the car-oriented zone. In the analyses, we examined the car-oriented zone (types of urban zones = 7), public transport zone (=4, 5, and 6), pedestrian zone (=1 and 3) and pedestrian zone of a sub-centre (=2) (see Fig. 2).

### Pedestrian and cycling network

Pedestrian and cycling networks were examined using Digiroad 2014 that is maintained by Finnish Transport Agency [41]. For analyses, we calculated the proportion of motorways, cycling and pedestrian roads as well as the proportion of other roads within a 250 m x 250 x spatial unit. A summary variable of cycling and pedestrian network variable was created from the indicators (see Table 1) including categories of 1) “no cycling or pedestrian networks”, 2) “roads that allow cycling and walking”, 3) “Less than 30 % of pedestrian and cycle lanes”, 4) “30^−^50 % of pedestrian and cycling lanes” and 5) “More than 50 % of pedestrian and cycling lanes” of the total proportion roads in the spatial unit. In these analyses, we examined the pedestrian and cycling network within 500 metres of the home location.

### Background and confounding variables

*Age* was limited to working age and divided into three groups: 30–44, 45–54 and 55–64. *Gender* (men vs. women) was adjusted. *Education* was divided into three categories: “low education”, “middle education” and “high education”. *Leisure-time physical activity* was assessed with the question “How much do you exercise and exert yourself physically during your leisure time?” The response alternatives were 1) “In my leisure time, I read, watch TV and do other minor activities that do not strain me physically”, 2) “In my leisure time, I walk, cycle and move in other ways ≥4 h/week”, 3) “In my leisure time, I exercise at least 3 h per week” and 4)”In my leisure time I practice regularly several times per week for a competition (answer this if you train full time for competition sports)”. *Occupational physical activity* was assessed with a question “How strenuous is your job physically” with the response alternatives 1) “In my job, I mainly sit and do not walk much”, 2) “I walk quite a bit in my job, but I do not need to lift or carry heavy things”, 3) “In my job I need to walk or lift quite a lot or climb stairs or walk uphill” and 4) “My job is heavy physical labour, and I have to lift or carry heavy items, dig, shovel, pound or do some other heavy labour”. For analyses, these were divided into “physically inactive” job (responses = 1) or “physically active” job (=2, 3 and 4).

### Statistical analyses

The sampling design and non-responses were accounted for in analyses using linearisation-based methods [42], with post-stratification weighting [33] and inverse probability weighting methods [43]. Based on these methods, post-stratification weights were produced and utilised in estimating the characteristics of the study population based on commuting physical activity and calculating odds ratios (OR) and 95 % confidence intervals (95CI) for commuting physical activity with survey logistic regression. All the statistical analyses were performed with STATA 11.2 software [44] using survey data analysis tools and population weights. The survey logistic regression modelling was conducted sequentially: first we adjusted for age, gender and environmental factors; then we adjusted for education, leisure-time and occupational physical activity.

In order to examine whether the association between environmental factors and commuting physical activity might differ between the individuals, we conducted following interaction analyses using survey logistic regression: individual factors*(recreational green space, travel-related urban zones or cycling and pedestrian network), and travel-related urban zones*(recreational green space or cycling and pedestrian network).

## Results

### Characteristics of the study population by commuting physical activity

The characteristics of the study population and associations with commuting physical activity are shown in Table 2. 55 to 64 year-old respondents reported more physical activity while commuting compared to the other age-groups. Women and those with high education reported commuting physical activity more often than men and those with low education. Those who were physically active in their leisure time or had a physically inactive job were also physically active while commuting. Respondents who lived in an area with less than 50 % of recreational green space and more than 200 metres proximity from green space were physically active while commuting. Moreover, over half of those respondents who lived in a pedestrian zone or in a pedestrian zone of a sub-centre reported being physically active while commuting. The high proportion of cycling and pedestrian lanes within the living environment was also associated among respondents with physical activity while commuting.

**Table 2:** Characteristics of the study population by commuting physical activity (*n* = 2 098)*

	Commuting physical activity			
	*n*	Inactive (*n* = 1234)	Active (*n* = 855)	*p*-value
Age groups		%	%	<0.05
30–44	783	61	39	
45–54	727	62	38	
55–64	579	54	46	
Gender				<0.001
Men	955	66	34	
Women	1134	53	47	
Education				<0.01
Low	197	58	42	
Middle	752	65	35	
High	1140	56	44	
Leisure-time physical activity				<0.001
reading, watching TV, and other minor activities	535	71	29	
walking and cycling more than 4 h per week	1023	53	47	
exercise at least 3 h per week	486	59	41	
practice competitive sports several times per week	45	55	45	
Occupational physical activity				0.05
physically active job	1064	62	38	
physically inactive job	949	57	43	
Proportion of green space^a^ (%)				<0.001
0	1234	65	35	
1–24.9	291	44	56	
25–49.9	510	54	46	
50+	54	66	33	
Distance to green space^b^ (in metres)				<0.05
0–199	924	61	38	
200+	1165	55	45	
Travel-related urban zones^c^				<0.001
car-oriented zone	1065	64	35	
public transport zone	577	53	47	
pedestrian zone	130	45	55	
pedestrian zone of a sub centre	317	34	66	
Proportion (%) cycling and pedestrian networks^d^				<0.001
No cycling or pedestrian networks	1236	65	35	
Other roads that allow cycling and walking	71	73	27	
Cycling and pedestrian lanes <30 %	235	53	47	
Cycling and pedestrian lanes 30–50 %	347	51	49	
Cycling and pedestrian lanes >50 %	200	43	57	

* ‘Active’ include those who engage physical activity while commuting; while ‘inactive’ include those who use public transport or car to commute

a: Proportion of green space (min 1.5 ha) around 500-metre buffer from home location, Corine Land Cover 2006

b: Euclidean distance (in metres) to the closest green space (min 1.5 ha), Corine Land Cover 2006

c: The main travel-related urban zone around 500-metre buffer from home location, Travel-related urban zones 2010

d: Proportion of cycling and pedestrian lanes from all roads around 500-metre buffer from home location, Digiroad 2014

Distribution of the examined variable (n), survey prevalence (%) and Pearson *p*-value from group differences in commuting physical activity (*p*-value)

### Contribution of travel-related urban zones, green space and cycling and pedestrian networks with commuting physical activity – a logistics regression analysis (Table 3)

After adjusting for age and gender, those respondents living in a pedestrian zone (Model 2: Odds Ratio = 1.88; 95 % Confidence Interval = 1.24–2.86) and in the pedestrian zone of a sub-centre (2.28; 1.23–4.21) were more likely to be physically active while commuting compared to those living in a car-oriented zone. The contribution of pedestrian zone and the pedestrian zone of a sub-centre on commuting physical activity was slightly attenuated but remained statistically significant after adjusting for proportion of cycling, education, leisure-time and occupational physical activity (in Model 5). Living in a public transport zone also contributed to a higher likelihood of physical activity while commuting, but the contribution attenuated and was not statistically significant after adjusting for the proportion of the cycling and pedestrian network (in Model 2).

**Table 3:** The contribution of travel-related urban zones, cycling and pedestrian network and green space with commuting physical activity among employed adults

	Model 1: age-adjusted		Model 2: Model 1 + travel-related urban zones + cycling and pedestrian network			Model 3: Model 2 + proportion of and distance to green space			Model 4: Model 3 + education			Model 5: Model 4 + leisure time & occupational physical activity		
	OR	95CI	OR	95CI	p*	OR	95CI	p*	OR	95CI	p*	OR	95CI	p*
Age-group (years)					<0.01			<0.01			<0.01			<0.01
30–44	1		1			1			1			1		
45–54	0.96	0.78–1.19	1.03	0.83–1.28		1.03	0.83–1.28		1.04	0.84–1.29		1.02	0.82–1.28	
55–64	1.31	1.47–2.10	1.40	1.12–1.76		1.40	1.11–1.75		1.41	1.12–1.79		1.41	1.12–1.77	
Gender					<0.001			<0.001			<0.001			<0.001
ref–men	1.83	1.52–2.19	1.85	1.55–2.22		1.86	1.55–2.23		1.81	1.51–2.18		1.80	1.49–2.17	
Travel-related urban zones^a^					<0.01			<0.01			<0.01			<0.01
car-oriented zone	1		1			1			1			1		
public transport zone	1.65	1.32–2.08	1.12	0.76–1.64		1.09	0.74–1.61		1.09	0.74–1.61		1.06	0.72–1.57	
pedestrian zone	2.41	1.75–3.31	1.88	1.24–2.86		1.72	1.12–2.62		1.69	1.10–2.58		1.63	1.06–2.51	
pedestrian zone of a sub centre	3.36	2.03–5.55	2.28	1.23–4.21		2.16	1.16–4.01		2.23	1.20–4.13		2.03	1.09–3.80	
Proportion (%) cycling and pedestrian networks^b^					<0.05			<0.01			<0.01			<0.01
no cycling or pedestrian roads	1		1			1			1			1		
other roads that allow cycling and walking	0.69	0.40–1.22	0.66	0.37–1.15		1.28	0.58–2.78		1.27	0.58–2.74		1.23	0.56–2.69	
Cycling and pedestrian lanes <30 %	1.67	1.26–2.21	1.27	0.88–1.85		2.25	1.25–4.06		2.21	1.23–3.96		2.26	1.25–4.09	
Cycling and pedestrian lanes 30–50 %	1.87	1.47–2.39	1.46	1.00–2.14		2.65	1.45–4.86		2.55	1.40–4.63		2.65	1.45–4.8	
Cycling and pedestrian lanes >50 %	2.30	1.69–3.14	1.76	1.10–2.80		3.12	1.63–5.99		2.96	1.55–5.66		3.28	1.71–6.31	
Proportion of green space^c^	1.23	1.11–1.35				0.75	0.57–0.97	<0.05	0.73	0.57–0.95	<0.05	0.73	0.57–0.94	<0.05
Distance (in metres) to green space^d^								ns						
0–199	1					1								
200+	1.35	1.10–1.65				0.91	0.67–1.23							
Education														
Low	1								1		ns			
Middle	0.95	0.68–1.32							0.89	0.63–1.24				
High	1.36	1.00–1.87							1.05	0.75–1.46				
Leisure time physical activity														<0.001
reading, watching TV, and other minor activities	1											1		
walking and cycling more than 4 h per week	2.14	1.71–2.67										2.10	1.67–2.64	
exercise at least 3 h per week	1.60	1.26–2.07										1.54	1.18–2.02	
practice competitive sports several times per week	1.57	0.82–3.03										1.94	1.00–3.80	
Occupational physical activity														ns
Physically inactive job	1											1		
Physically active job	0.78	0.65–0.93										0.87	0.72–1.05	

Analyses conducted among working aged Finns (total n = 2 089) using stepwise survey logistic regression, odds ratios (OR) and 95 % confidence intervals (95CI)

*p-value from Wald-test for inclusion of contributing factor in Model X having a statistically significant effect on commuting physical activity

a: The main travel-related urban zone around 500-metre buffer from home location, Travel-related urban zones 2010

b: Proportion of cycling and pedestrian lanes from all roads around 500-metre buffer from home location, Digiroad 2014

c: Proportion (%) of green space (min 1.5 ha) around 500-metre buffer from home location, Corine Land Cover 2006

d: Euclidean distance (in meters) to the closest green space (min 1.5 ha), Corine Land Cover 2006

The higher the proportion of cycling and pedestrian networks the higher the likelihood for physical activity while commuting. The likelihood of commuting physical activity was over three times higher when proportion of cycle and pedestrian lanes was 50 % or over in the road network compared to when there were no cycling or pedestrian lanes (Model 5: 3.12; 1.63–5.99) after adjusting for all environmental factors. The contribution of a cycling and pedestrian network to commuting physical activity remained after adjusting for education, leisure-time physical activity and occupational physical activity.

A higher proportion of green space close to home contributed to a likelihood of physical activity while commuting in the age-adjusted model (Model 1: 1.23; 1.11–1.35). Longer distance to green space (200 metres or more) contributed to a higher likelihood of commuting physical activity (Model 1: 1.35; 1.10–1.65) compared with those living 0–199 metres from green space after adjusting for age. However, when both indicators of green space were simultaneously added to the model (in Model 3), the high proportion of green space close to the home location contributed negatively to the commuting physical activity (0.75; 0.57–0.97) after adjusting for other environmental factors. The contribution of the proportion of green space to commuting physical activity remained after adjusting for education, leisure-time physical activity and occupational physical activity (in Models 4 & 5).

### Interaction analyses between individual factors, environment and commuting physical activity

In order to examine whether association between environmental factors and commuting physical activity differed by individual and environmental factors, the following interaction analyses with logistic regression, odds ratios and *p*-values was carried out (Table 4). We utilized *p*-value < 0.10 as the level of statistical significance for the interactions.

**Table 4:** Interactions of the individual and environmental factors for commuting physical activity among employed Finns

	Commuting physical activity	
	OR	*p*-value
agegroup of 45–54 * pedestrian zone of sub centre	0.30	<0.10
women * public transport zone	1.62	<0.05
women * proportion of cycling and pedestrian lanes more than 50 %	1.77	<0.10
leisure-time physical activity * proportion of green space is 25–49.9 %	1.53	<0.10
travel-related urban zones*proportion of green space %	-	ns
travel-related urban zones*cycling and pedestrian network	-	ns
proportion of green space * cycling and pedestrian network	-	ns

Survey logistic regression, odds ratios (OR) and *p*-value for statistical sigfinicance (*p*-value < 0.10)

a: The main travel-related urban zone around 500-metre buffer from home location, Travel-related urban zones 2010

b: Proportion of cycling and pedestrian lanes from all roads around 500-metre buffer from home location, Digiroad 2014

c: Proportion (%) of green space (min 1.5 ha) around 500-metre buffer from home location, Corine Land Cover 2006

*: interaction between the examined variables

Those who were aged from 45 to 54 years and lived in a pedestrian zone of a sub-centre were less likely to be physically active while commuting. Women living in a public transport zone (OR = 1.62; *p*-value <0.05) or in an area with a proportion of cycle and pedestrian lanes of 50 % or more (1.77; *p* < 0.10) were more likely to be physically active while commuting. Moreover, those individuals who were physically active during their leisure-time and lived in an area with a proportion of green space of 25–49.9 % were more likely to be physically active while commuting (1.53; *p* < 0.10). Interactions between travel-related urban zones*proportion of green space, travel-related urban zones*cycling and pedestrian network and proportion of green space*cycling and pedestrian network showed no statistically significant differences for commuting physical activity.

## Discussion

This study is among the first to combine geographical information system data with a large population-based health examination data including commuting physical activity information. A high proportion of cycling and pedestrian network in the home neighbourhood and living in a pedestrian zone contributes to walking and cycling to and from work among employed Finns living in the five large cities in Finland. However, the high proportion of green space near the home environment contributes negatively to walking and cycling to and from work. Moreover, individuals aged between 45 and 54 years were less likely to walk or cycle to and from work when living in the pedestrian zone of sub-centre. Women living in public transport zone or in an area with a high proportion of cycle paths and footpaths were more likely to be physically active while commuting. Individuals who were physically active during leisure-time and living in an area with a high proportion of recreational green space were also more likely to be physically active while commuting.

In our study, a high proportion of cycling and pedestrian networks contribute positively among employed Finns to walking and cycling to and from work. This is in accordance with previous studies, where a high density of cycle paths [15, 18] and other roads suitable for cycling and walking [27] have shown a positive contribution with transport-related physical activity, walking and cycling. In our study, the availability of cycling and pedestrian networks had a strong and independent influence on physical activity while commuting. This suggests that a good pedestrian infrastructure may efficiently promote physical activity of employed population while commuting [25, 26].

Environmental factors alone might not, however, promote walking and cycling to and from work among individuals, especially men, living in pedestrian zones of sub-centres. Based on our additional analyses with a smaller physical activity sub-sample (data not shown), over half of those respondents living in the pedestrian zone of a sub-centre reported over 10 kilometres distance to work. As the average commuting distance from home to work was 14 km in 2010 [9], the distance of 10 kilometres might already be quite discouraging for many people to choose walking or cycling instead of public transport or their own car to go to work. Moreover, one could speculate that, for some individuals who travel by public transport to work the proportion of pedestrian roads and cycling roads around their home neighbourhood or around their work location is not relevant, if they do not have other alternatives to travel to work.

Based on our study, 45–54-year old individuals were more likely to be physically inactive while commuting compared to younger age-groups. Based on our earlier study, especially women with children reported a lack of time as a key barrier to leisure-time physical activity [45]. Therefore, a lack of time might be a relevant barrier to daily commuting physical activity as well, as some parents might have to transport children to day-care or school by car. Our preliminary analyses (data not shown) also support this, as those women and men living together with children were less likely to be physically active while commuting. In the first stages of statistical analyses (data not shown), we also examined health and chronic conditions as well as social support as potential co-factors for walking and cycling to and from work, but they did not show a statistically significant contribution to cycling and walking after the factors used in current final model.

The importance of good access to a green space for choosing walking and cycling to travel to and from work is inconsistent and very few studies have been conducted in European context [15, 18]. Our results suggest that the proximity of and a high proportion of green space close to home might have a negative contribution to commuting physical activity among the working population in a Finnish context. One contextual reason behind this might be that residential areas outside the core urban areas in Finland are greener (including natural forests, and larger recreational areas) than elsewhere in Europe. People living there generally have better access to green space, but that green space might not be suitable for commuting physical activity compared to the urban forests, parks or other green space inside densely built urban areas. In addition, individuals living in residential areas outside the core urban areas may prefer to utilize car or public transport for commuting to work due to long distances. This was also observed in our additional analyses with a smaller physical activity sub-sample (only half of the total sample), showing that people living in the area with a high proportion of green space (25 % or more) were likely to report longer commuting distances to work. Moreover, a very high proportion of green spaces (50 % or more) coincide with car-oriented urban zones, which are mainly situated outside Finnish urban core areas.

Some studies have suggested that the association between environmental factors and physical activity might differ by socioeconomic position, social support and by neighbourhood [31, 46, 47]. This study was only partly able to repeat these results for commuting physical activity. We conducted several interaction analyses between individual, social and environmental factors and commuting physical activity. Social support or education showed no statistically significant interactions with environment and commuting physical activity (data not shown). The interaction analyses of the proportion of green space, travel-related urban zones, and the proportion of cycling and pedestrian network locations showed a dependence on commuting physical activity, but the results were not statistically significant.

Working-age women were more likely to be physically active while commuting than working-age men. The sociodemographic distribution of the role of environment to commuting physical activity among adults is scarce [1, 6]. However, from studies examining leisure-time walking and cycling it has been suggested that men were found to be more likely to undertake recreational walking in their residential neighbourhood [14]. In another study [16], women were more likely to be physically active in their residential neighbourhood. Based on interaction analyses, one reason for this might be that women use cycling and pedestrian networks for commuting physical activity more frequently than men. Moreover, based on our previous studies, working-aged Finnish women and those with high education are more likely to be health-oriented and practise healthy and physically active lifestyles compared to working-aged men and those with low education [32, 48].

The analyses also demonstrated that individuals who were physically active during their leisure-time and lived in an area with a high proportion of recreational green space were also more likely to walk and cycle to and from work. Theoretical perspectives of healthy lifestyles suggest that an individual might be physically active and practice multiple other healthy behaviors as well as also choose to live in health promoting environment [5, 49, 50]. For example, those who work in physically strenuous working conditions might be less motivated to be physically active [30, 51]. Similarly, recent studies have suggested that individuals who show a basic interest in physical activity are more likely to utilize green space, cycling and pedestrian networks to be physically active while commuting [24, 25].

### Methodological considerations

Some limitations should be remembered when interpreting the results of this study. This study was only able to examine associations between environmental factors and commuting physical activity using a cross-sectional study design and was not able to determine causal mechanisms behind environmental determinants for commuting physical activity. In addition, individuals might have various preferences for choosing a home environment; for example, physically active individuals probably choose their home location according to the ability to carry out their physically active lifestyle, and changes in the individual’s neighbourhoods, such as new walking and cycling routes, might affect the individual’s physical activity behaviour [20, 26].

The strength of the current study was, however, that it is based on a large population-based health examination survey, Health 2011, which included a representative random sample of women and men aged 30 years and over drawn from the national population registers [32]. The general response rate of 72.5 % can be seen as relatively good, but lower participation rates were observed among men, younger age groups and those with lower educational attainments [52]. Moreover, we were able to investigate a large variety of questionnaire, interview, and measured information on physical activity behaviours, sociodemographic and health of the residents living in the core urban areas of the five largest Finnish cities (Helsinki, Turku, Tampere, Kuopio and Oulu) and their sub centres (see Fig. 1, right side). However, although a large amount of information on physical activity, health and welfare as well as background information was collected and examined in this study, not all potentially relevant environmental or individual level factors influencing commuting physical activity were included in the Health 2011 health examination study, such as safety and safety routes to work, car or bicycle ownership [1, 6, 53].

As the information on green space, travel-related urban zones and survey data was from different time periods (CORINE Land Cover in 2006, Health 2011 study in 2000–2011, Travel-related urban zones/YKR in 2010, Digiroad 2014), this may have influenced our results. Collection of environmental information using geographical information systems can take general several years and even more before that data is ready and available for research purposes. However, as this was the latest official GIS data available at the time of the study and the environmental variation can be assumed to be very low, this would not dramatically influence our results. Moreover, the same GIS data and indicators of green space and travel-related urban zones are being used by urban planners in many Finnish cities [36, 40].

Commuting physical activity was surveyed with a self-administrated questionnaire. Our question of commuting physical activity measures the average situation and the main mode of physical activity while commuting to work. It, therefore, only distinguishes those who are physically active while commuting to and from work from those who are physically inactive. It might in some cases underestimate physical activity among individuals who are, for example, 10 min physically active while walking to bus stop and then ride 30 min on a bus as a main mode of travel to work. To verify our results regardless of the cut-off point of commuting physical activity, sensitivity analyses with a cut-off point of 15 or more minutes per day of physical activity while commuting to work were conducted. The results using a different cut-off point demonstrated similar results compared to the cut-off point any physical activity while commuting used in the results of this study. Questionnaires and simple questions of commuting physical activity are a common way of measuring physical activity in large population studies [54]. In general, physical activity assessed with questionnaires very strongly predicts accelerometer-based physical activity among adults [55, 56]. Our physical activity questionnaire has been found to strongly predict morbidity and mortality [34, 35]. Future studies should, however, examine in more detail routes of commuting physical activity utilizing objective accelerometers, global positioning systems and mobile applications as well as examine environmental determinants during the routes from home to work. Moreover, determinants for environment specific physical activity, such as physical activity in forests, parks or on urban streets should be examined further.

In the Finnish context, Euclidian distance to green space used in this research does not dramatically differ from the network distance, as the urban form rarely sets limitations on access to green space or on the pedestrian infrastructure in urban areas. However, in some cases there may be a large green space close to home but not in the direction of commuting. Moreover, in some urban areas the workplace might not be located in a pedestrian zone or a public transport zone, and thus an individual may have to choose not to be physically active, i.e. to take a car, while commuting.

## Conclusions

The current political agenda aims to promote active environments and physical activity as a part of daily life, but research has so far provided mixed results between environmental factors and commuting physical activity. Moreover, as previous research has mainly been conducted outside Europe, these findings cannot simply be transposed to the European context where urban form and urban green space are quite different. This study was both innovative in combining geographical information system data onto a large population-based health examination data and was policy-relevant. Based on our results among employed Finns, some policy-relevant suggestions can be made. Firstly, a good pedestrian and cycling infrastructure may play an important role in promoting commuting physical activity among the employed population, regardless of educational background, leisure-time and occupational physical activity. Secondly, distance to and proportion of green space near the home location may not be sufficient to initiate commuting physical activity. Third, age and gender variation in the environmental factors, such as cycling and walking networks and travel-related urban zones, on commuting physical activity should be taken into account when planning transport and physical activity policies.
